# Differential effects of propofol and dexmedetomidine on explicit and implicit memory after sedation: a retrospective study

**DOI:** 10.3389/fmed.2026.1871238

**Published:** 2026-07-06

**Authors:** Bojun Zhang, Fang Jia, Ping Li

**Affiliations:** Department of Anesthesia, Tianjin Hospital, Tianjin, China

**Keywords:** calm, dexmedetomidine, postoperative cognitive impairment, propofol, retrospective cohort study

## Abstract

**Objective:**

To compare the effects of dexmedetomidine and propofol on early postoperative cognitive function and safety in patients undergoing short-term surgical sedation.

**Methods:**

This retrospective cohort study included 295 adult patients who received intravenous sedation during non-cardiac surgery (January 2023–January 2025). Patients were divided into dexmedetomidine (*n* = 150) and propofol (*n* = 145) groups. Postoperative day 1 outcomes included explicit memory (RAVLT delayed recall) and processing speed (picture naming reaction time). Perioperative hemodynamics, recovery time, and adverse events were recorded.

**Results:**

On postoperative day 1, the dexmedetomidine group showed higher RAVLT delayed recall scores (8.08 ± 3.06 vs. 7.24 ± 2.82, *p* = 0.015) but longer picture naming reaction time (881.42 ± 125.34 ms vs. 847.53 ± 118.76 ms, *p* = 0.037) compared to propofol. Median recovery time was longer with dexmedetomidine (18.00 vs. 14.00 min, *p* < 0.001). Dexmedetomidine was associated with more bradycardia requiring intervention (*p* = 0.034), while propofol had more injection pain (*p* < 0.001). No significant differences were found in hypoxemia or hypotension (*p* > 0.05). Multivariate analysis identified dexmedetomidine use as an independent factor for higher postoperative RAVLT scores (*p* = 0.007).

**Conclusion:**

In this short-term sedation cohort, dexmedetomidine was associated with better explicit memory but slower processing speed and longer recovery versus propofol, with distinct safety profiles. The clinical significance of these small cognitive differences remains uncertain, and prospective validation is needed.

## 1 Introduction

Postoperative cognitive impairment (POCD) is a common complication after surgery, which is closely related to the selection of anesthesia and sedative drugs, and has become a research hotspot in perioperative medicine (1, 2). Among numerous intravenous sedatives, propofol and dexmedetomidine are widely used due to their unique pharmacological properties. Propofol, as a classic gamma aminobutyric acid (GABA) receptor agonist, can rapidly induce loss of consciousness, but its potential widespread inhibitory effect on cognitive networks has attracted sustained attention (3). In contrast, dexmedetomidine, as a highly selective *α* 2-adrenergic receptor agonist, is believed to induce a “sleep like” sedative state that is closer to natural sleep in neurophysiology (4). The fundamental difference in this mechanism naturally raises a core clinical question: is there a fundamental difference in the impact of these two drugs on patients’ early postoperative cognitive function? Previous studies have not provided a unified conclusion on this issue (5, 6). Partial basic research suggests that dexmedetomidine may alleviate propofol induced neuronal apoptosis and inflammatory response, thereby demonstrating potential neuroprotective value (7). However, clinical evidence shows considerable heterogeneity (8). Some observational studies suggest that monotherapy sedation with dexmedetomidine may be associated with better prognosis in mechanically ventilated patients in the intensive care unit (ICU) (9). On the other hand, studies have also found that in patients undergoing conscious craniotomy surgery, the combination of dexmedetomidine and propofol may actually prolong the response time of cognitive tasks, suggesting that it may have adverse effects on information processing speed (10).

Although existing research has accumulated some evidence, there are still several obvious limitations that cannot be ignored (11). Firstly, most clinical studies evaluate cognitive function as a general entity, lacking detailed differentiation and synchronous examination of different cognitive dimensions such as explicit memory consolidation and implicit information processing speed (12). However, neuropharmacological studies have already revealed that there is regional specificity in the mode of action of different sedatives on the brain neural network (13). Secondly, existing studies often focus on one aspect of outcomes (such as delirium incidence or short-term neuropsychological test scores) when comparing two drugs, and fail to comprehensively weigh cognitive outcomes with drug specific safety profiles (such as bradycardia associated with dexmedetomidine and injection pain associated with propofol) within the same framework (14). This separated evaluation is difficult to guide clinical doctors in making individualized drug choices when facing specific patients (15). Finally, many studies have limited sample sizes or are conducted in highly specific patient populations, such as those undergoing cardiac surgery or neurosurgery, which limits the generalizability of their conclusions. Therefore, there is an urgent need for a study to be conducted in a broader population of non-cardiac surgery patients to systematically compare the comprehensive effects of these two commonly used sedatives on multidimensional cognitive function and perioperative safety.

Based on the research gap mentioned above, this study aims to conduct a retrospective cohort analysis to explore the differences in the effects of dexmedetomidine and propofol on different cognitive dimensions (explicit memory and processing speed) in the early postoperative period of non-cardiac surgery patients undergoing intraoperative sedation, and comprehensively evaluate their related recovery quality and adverse reaction spectrum. We hope that the research results can provide more targeted evidence-based medicine for the rationalization and personalized selection of sedatives in clinical anesthesia practice.

## 2 Data and methods

### 2.1 General information

**Figure 1:**
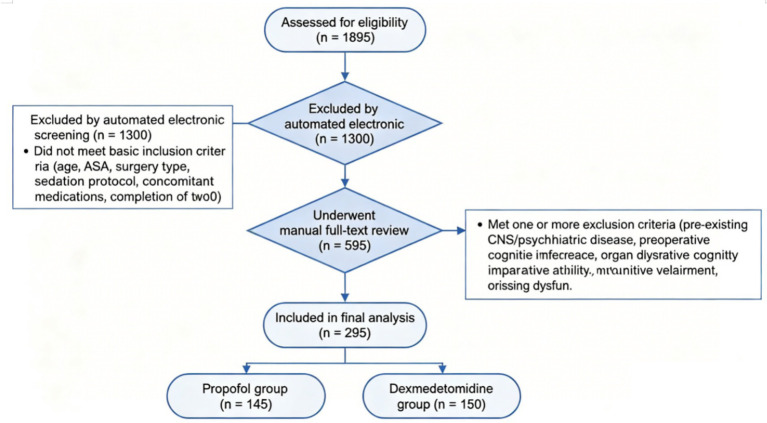
Patient study flowchart. Patient study flowchart illustrating the identification, screening, eligibility assessment, and final inclusion of patients in the propofol and dexmedetomidine groups. Reasons for exclusion are summarized at each step.

This study is a single center, retrospective cohort study. The research plan has been approved by the Medical Ethics Committee of our university. Due to the retrospective nature of the study and strict protection of patient privacy, the ethics committee waived the requirement to obtain informed consent from patients. The research data is sourced from our hospital’s anesthesia clinical information system, electronic medical record system, and neuropsychological evaluation database. During the study period, a hospital-wide quality improvement initiative was in effect, under which all adult patients receiving intravenous sedation in the PACU or ICU underwent a standardized neurocognitive assessment battery approximately 24 h after sedation as part of routine postoperative monitoring, unless clinically contraindicated. These assessments were conducted by the dedicated neuropsychological evaluation unit and were not specifically planned for research purposes. The research period is defined as January 1, 2023 to January 31, 2025. During this period, a total of 1895 adult patients received planned intravenous sedation under intraoperative or postoperative monitoring. Through the pre-set electronic screening process, a total of 295 patients were ultimately included in the final analysis. According to the main intravenous sedatives used during sedation maintenance, patients were divided into two groups: propofol group (145 cases) and dexmedetomidine group (150 cases). The sample size of this retrospective study was not pre calculated, but based on all available cases that met strict inclusion and exclusion criteria during the study period, which is consistent with the exploratory and hypothesis generated retrospective study design. The screening and inclusion process of the two groups of patients followed clear steps: firstly, all cases of sedation using propofol or dexmedetomidine were exported from the information system; Secondly, using automated scripts combined with manual verification to screen out records that do not meet basic criteria such as age, surgical type, and medication combination; Finally, two independent researchers reviewed the complete medical records of the remaining cases and confirmed them strictly based on inclusion and exclusion criteria. In case of disagreement, a third senior researcher arbitrated (Figure 1).

### 2.2 Inclusion and exclusion criteria

Inclusion criteria:

(1) The age range is between 18 and 65 years old.
(2) The American Society of Anesthesiology (ASA) classifies it as grade I or II.
(3) Received general anesthesia for various elective non cardiac and non-intracranial surgeries, and received continuous intra and postoperative intravenous sedation, with the goal of promoting mechanical ventilation tolerance or relieving agitation.
(4) The intravenous sedation regimen is single drug continuous infusion: propofol (infusion rate ≥ 4 mg/kg/h) or dexmedetomidine (infusion rate ≥ 0.2 *μ* g/kg/h), and the continuous infusion time is not less than 30 min.
(5) During the sedation period and within 24 h after its completion, no other drugs that may significantly affect cognitive function were used, such as benzodiazepines, ketamine, anticholinergic drugs, or antipsychotic drugs.
(6) Within 24 h (± 2 h) after sedation, the standardized postoperative neurocognitive function assessment package was completed by our hospital’s neuropsychological evaluation room.

Exclusion criteria:

(1) There is a clear history of central nervous system diseases (such as dementia, epilepsy, stroke, severe traumatic brain injury), a history of mental illness (such as schizophrenia, major depression), or a history of substance abuse (alcohol or illegal drugs).
(2) In preoperative baseline assessment, a Mini Mental State Examination (MMSE) score below 24 indicates the presence of preoperative cognitive impairment.
(3) Severe combined liver and kidney dysfunction is defined as levels of aspartate aminotransferase (AST) or alanine aminotransferase (ALT) exceeding three times the upper limit of normal, or estimated glomerular filtration rate (eGFR) < 60 mL/min/1.73 m ^2^.
(4) Severe hemodynamic instability requiring continuous infusion (>10 min) of vasoactive drugs (such as norepinephrine) during surgery or sedation, or clinically significant hypotension (mean arterial pressure<55 mmHg) or bradycardia (heart rate<45 beats/min).
(5) Due to surgical complications, severe bleeding, or other reasons, patients may be directly transferred to the ICU after surgery and are expected to receive mechanical ventilation for more than 48 h, or require a second surgery.
(6) Medical records are missing key information, including specific dosage, duration, hemodynamic records, or neuropsychological evaluation results of sedatives.

### 2.3 Research methods

(1) *Sedation plan*: Sedation management for all patients is carried out by anesthesiologists or ICU physicians according to the routine of the department at that time. The target for sedation depth is usually set at a Ramsay sedation score of 3–4 (patients have a clear response to instructions but are drowsy). Patients in the propofol group received intravenous infusion of propofol (Diprolimus, AstraZeneca). The administration mode includes Target Controlled Infusion (TCI), with an effect chamber target concentration set at 1.0–3.0 *μ* g/mL, or manual infusion based on body weight, with a maintenance rate adjusted within the range of 4–12 mg/kg/h. Patients in the dexmedetomidine group received intravenous infusion of dexmedetomidine hydrochloride (Ai Beining, Jiangsu Hengrui Pharmaceutical). The standard protocol is to administer a loading dose of 1.0 *μ* g/kg, which should be administered intravenously within 10 min, followed by continuous infusion at a rate of 0.2–0.7 μ g/kg/h. If significant bradycardia (heart rate<50 beats/min) or hypotension (systolic blood pressure<90 mmHg) occurs during the loading dose infusion or maintenance period, the infusion should be paused or the infusion rate should be reduced and recorded as an adverse event.
(2) *Data extraction*: Using a uniformly designed electronic data collection form, two trained anesthesia graduate students independently extracted data from the hospital information system. The extractors were blinded to the specific study hypotheses but were aware of the drug names as recorded. Any discrepancies were resolved through discussion with a third senior investigator by referring to the original medical records. The extracted content includes demographic data (age, gender, body mass index), surgical and anesthesia details (surgical type, duration, anesthetic drugs), sedation related parameters (drug name, total dose, infusion duration, loading dose usage), perioperative physiological parameters, and neuropsychological evaluation results. After the extraction is completed, cross check and resolve any inconsistencies through consultation with the original medical records.
(3) *Grouping definition*: The main sedative drugs continuously administered during sedation maintenance period are used as the grouping basis. For patients who temporarily add a single dose of propofol (dose ≤ 0.5 mg/kg) to rapidly deepen sedation during infusion of dexmedetomidine, they are still classified as the dexmedetomidine group. But if the record shows continuous infusion of two drugs in combination, the case is excluded.

### 2.4 Observation indicators

(1) Main outcome measures:

a. *Explicit memory function*: The Rey Auditory Verbal Learning Test (RAVLT) Chinese version’s “Delayed Recall” score was used for evaluation. The test is conducted 24 h after the end of sedation. The evaluator will first read out 15 random commonly used vocabulary words and ask the patient to immediately recall it, repeating it 5 times (T1-T5). After a 30 min interval, patients are asked to freely recall as many words as possible without reading the word list again, and record the number of correctly recalled words (0–15 points). This score is a core indicator for evaluating the ability to consolidate episodic memory.
b. *Implicit memory function*: evaluated through the “Picture Naming Reaction Time” task. 24 h after sedation, use E-Prime 3.0 software (Psychology Software Tools, United States) to present 40 color object images (such as apples and cars) on a laptop. Before the formal task, each participant completed a practice block consisting of 5 trials using different images to ensure comprehension of the task instructions. Subsequently, 40 color object images (e.g., apple, car) were presented. Record the reaction time (in milliseconds) from image presentation to correct patient naming. Calculate the average reaction time for all correctly named trials. The shorter the reaction time, the better the triggering effect of procedural memory or perception, reflecting implicit memory function.

(2) Secondary outcome measures:

c. *Sedation quality*: Record the proportion (%) of time during sedation that reaches the target sedation depth (Ramsay score 3–4). Simultaneously record the incidence of body movements or coughing events that require intervention during the sedation process.
d. *Hemodynamic stability*: Extract the mean arterial pressure (MAP) and heart rate (HR) at baseline before the start of sedation, 10 min, 30 min after the start of sedation, and four time points before the end of sedation. Calculate the maximum fluctuation amplitude (*Δ* maximum) of each group’s MAP and HR relative to the baseline. Record the number of hypotension or bradycardia events that require medication intervention (such as the use of ephedrine or atropine).
e. *Respiratory safety*: Record the lowest value of peripheral oxygen saturation (SpO2) during sedation. Count the number of patients with hypoxemia (defined as SpO2 < 90% and lasting for more than 30 s).
f. *Recovery feature*: Record the time (in minutes) from the cessation of sedative drug infusion to the patient reaching an Aldrete score of ≥ 9, as the awakening time. The Aldrete score evaluates recovery from five aspects: activity, respiration, circulation, consciousness, and blood oxygen saturation, with a total score of 10 points.
g. *Early postoperative cognitive function*: Screening will be conducted using the MMSE score on the first day after surgery. Record the proportion of patients in both groups whose MMSE scores have decreased by ≥ 2 points from preoperative baseline.
h. *Adverse events*: The system collects and records all adverse events that occur during sedation and within 24 h thereafter, including nausea and vomiting, headache, dry mouth (commonly with dexmedetomidine), injection pain (commonly with propofol), and any newly developed arrhythmias.

### 2.5 Statistical methods

All statistical analyses were conducted using IBM SPSS Statistics software (version 27.0, Armonk, NY). The continuity variable is first evaluated for normality using the Shapiro Wilk test. Variables that follow a normal distribution are expressed as mean ± standard deviation, and independent sample t-test is used for inter group comparison; Non normally distributed variables are represented by median (interquartile range), and Mann Whitney U test is used for inter group comparisons. Categorical variables are expressed as examples (percentages), and between group comparisons are conducted using chi square test or Fisher’s exact probability method (when the expected count is less than 5). Given the non-randomized grouping in retrospective studies, in order to control for the influence of potential confounding factors on the primary outcome, an Analysis of Covariance (ANCOVA) will be conducted on the two main indicators of RAVLT delayed recall score and average response time for image naming. The pre-selected covariates include age, preoperative MMSE baseline score, and surgical duration. The analysis results will report the adjusted least squares mean and its 95% confidence interval. All hypothesis tests are two-sided tests, with a *p*-value<0.05 indicating statistically significant differences. This study did not perform alpha error correction on the comparison of multiple secondary outcomes, therefore the analysis results for secondary outcomes should be considered exploratory and used to generate hypotheses for future prospective studies.

## 3 Result

### 3.1 Baseline characteristics

Table 1 shows that the baseline characteristics of the patients in the propofol group and the dexmedetomidine group, including age, gender, body mass index, ASA grade, type of operation, length of operation, amount of intraoperative bleeding, preoperative MMSE score, Ramsay sedation score, hypertension, diabetes and other complications, have no statistical difference (all *p* > 0.05), indicating that the two groups of patients are comparable before intervention. See Table 1.

**Table 1:** Comparison of baseline characteristics between the two groups.

Baseline characteristic	Propofol group (*n* = 145)	Dexmedetomidine group (*n* = 150)	Statistic (*t*/*χ^2^*)	*p*-value
Age (years, mean ± SD)	49.74 ± 12.38	51.26 ± 13.15	*t* = −1.021	0.308
Male [*n*(%)]	76 (52.41)	81 (54.00)	*χ^2^* = 0.074	0.786
Body mass index (kg/m^2^, mean ± SD)	24.51 ± 3.22	24.78 ± 3.46	*t* = −0.702	0.483
ASA class I [*n* (%)]	58 (40.00)	62 (41.33)	*χ^2^* = 0.054	0.817
Surgery type—orthopedic [*n*(%)]	42 (28.97)	45 (30.00)	*χ^2^* = 0.037	0.847
Surgery type—general surgery [*n*(%)]	53 (36.55)	54 (36.00)	*χ^2^* = 0.010	0.92
Duration of surgery (min, mean ± SD)	78.45 ± 24.67	81.20 ± 26.33	*t* = −0.935	0.35
Intraoperative blood loss (mL, mean ± SD)	145.30 ± 88.50	152.60 ± 92.40	*t* = −0.690	0.491
Preoperative MMSE score (mean ± SD)	28.12 ± 1.85	28.05 ± 1.92	*t* = 0.318	0.75
Preoperative ramsay score (mean ± SD)	2.15 ± 0.42	2.18 ± 0.44	*t* = −0.602	0.547
History of hypertension [*n* (%)]	38 (26.21)	42 (28.00)	*χ^2^* = 0.119	0.73
History of diabetes mellitus [*n* (%)]	22 (15.17)	25 (16.67)	*χ^2^* = 0.120	0.729
Smoking history [*n* (%)]	34 (23.45)	36 (24.00)	*χ^2^* = 0.012	0.912
Alcohol use history [*n* (%)]	29 (20.00)	31 (20.67)	*χ^2^* = 0.021	0.886
Time from sedation end to cognitive testing (hours)	20.82 ± 3.52	21.12 ± 3.23	*t* = −0.761	0.447
Duration of sedation (min)	82.54 ± 25.34	85.13 ± 27.02	*t* = 0.849	0.397

Comparison of baseline demographic, clinical, and perioperative characteristics between the propofol group and the dexmedetomidine group. Data are presented as mean ± SD, median (IQR), or *n* (%). P-values were obtained using independent samples t-test, Mann–Whitney U test, or chi-square test, as appropriate.

### 3.2 Primary endpoint results

Table 2 data shows that at the primary endpoint, the RAVLT delayed recall score of the dexmedetomidine group was significantly higher than that of the propofol group (*t* = −2.449, *p* = 0.015), but its image naming reaction time was also longer than that of the propofol group (*t* = −2.104, *p* = 0.037). In the secondary endpoint, the recovery time of the dexmedetomidine group was longer than that of the propofol group (*Z* = -4.038, *p* < 0.001), and the proportion of patients whose MMSE score decreased by ≥ 2 points from baseline on the first day after surgery was lower than that of the propofol group (*χ*^2^ = 4.334, *p* = 0.037). See Table 2.

**Table 2:** Comparison of primary and secondary endpoints between the two groups.

Outcome measure	Propofol group (*n* = 145)	Dexmedetomidine group (*n* = 150)	Statistic (*t*/*χ*^2^/Z)	*p*-value
Primary endpoints				
RAVLT delayed recall score (mean ± SD)	7.24 ± 2.82	8.08 ± 3.06	*t* = −2.449	0.015
Picture naming reaction time (ms, Mean ± SD)	847.53 ± 118.76	881.42 ± 125.34	*t* = −2.104	0.037
Secondary endpoints				
Proportion of time within target ramsay score (%, mean ± SD)	85.12 ± 12.34	82.65 ± 13.87	*t* = 1.188	0.236
Incidence of intra-sedation body movement/coughing events [*n* (%)]	12 (8.28)	8 (5.33)	*χ^2^* = 0.995	0.318
Emergence time (min, median [IQR])	14.00 (10.00, 18.00)	18.00 (14.00, 23.00)	*Z* = −4.038	<0.001
Lowest SpO₂ (%, Mean ± SD)	94.51 ± 3.42	95.12 ± 3.67	*t* = −1.498	0.135
Hypoxemia (SpO₂ < 90%) [*n* (%)]	11 (7.59)	7 (4.67)	*χ^2^* = 1.088	0.297
MMSE decline ≥2 points from baseline on POD1 [*n* (%)]	35 (24.14)	22 (14.67)	*χ^2^* = 4.334	0.037

### 3.3 Secondary endpoint results

The recovery time of the dexmedetomidine group was significantly longer than that of the propofol group (median 18.00 min vs. 14.00 min, *p* < 0.001). The proportion of early postoperative cognitive decline (MMSE decrease ≥ 2 points) was lower in the dexmedetomidine group (14.67% vs. 24.14%, *p* = 0.037).

### 3.4 Subgroup analysis results

**Figure 2:**
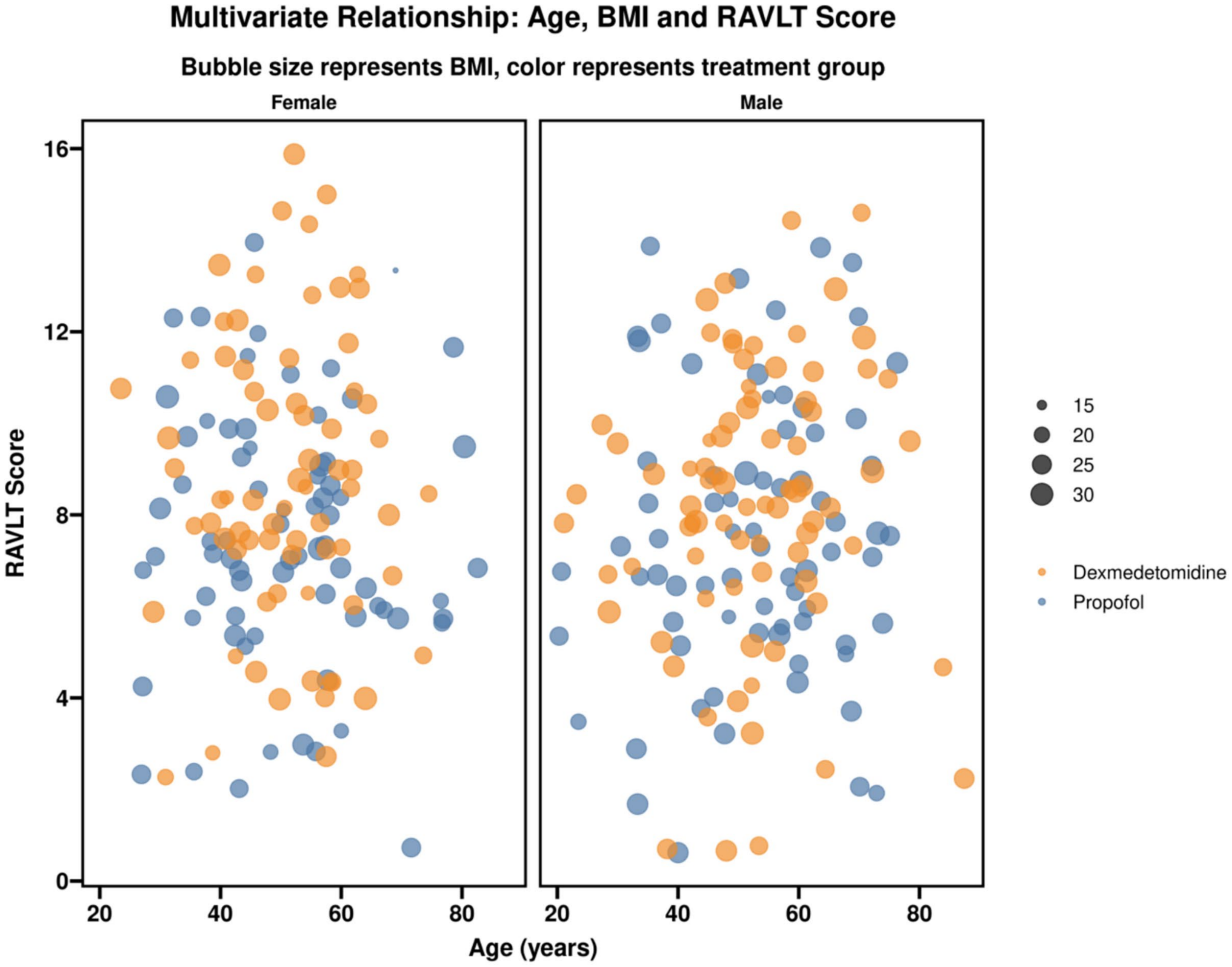
Intergroup differences in RAVLT delayed recall score. Subgroup differences in RAVLT delayed recall score between the propofol and dexmedetomidine groups stratified by age (<60 years, ≥60 years) and sex (male, female). Data are presented as mean ± SD. The *p*-values refer to the interaction effect.

The subgroup analysis in Table 3 shows that no significant interaction was observed between RAVLT delayed recall scores stratified by age (<60 years and ≥ 60 years) or gender (male and female) (interaction *p*-values of 0.241 and 0.368, respectively), indicating that age and gender did not significantly alter the effect of sedatives on delayed recall. See Table 3 and Figure 2.

**Table 3:** Subgroup analysis: intergroup differences in RAVLT delayed recall score.

Subgroup	Propofol group (mean ± SD)	Dexmedetomidine group (mean ± SD)	Mean difference (95% CI)	Interaction *p*-value
Age <60 years (*n* = 231)	7.35 ± 2.79	8.14 ± 3.02	−0.79 (−1.44, −0.14)	0.241
Age ≥60 years (*n* = 64)	6.82 ± 2.91	7.88 ± 3.21	−1.06 (−2.48, 0.36)	—
Male (*n* = 157)	7.18 ± 2.76	8.02 ± 3.10	−0.84 (−1.66, −0.02)	0.368
Female (*n* = 138)	7.31 ± 2.89	8.15 ± 3.02	−0.84 (−1.72, 0.04)	—

### 3.5 Safety results

Table 4 shows that there are differences in the spectrum of adverse events between the two groups. The incidence of injection pain in the propofol group was significantly higher than that in the dexmedetomidine group (*χ*^2^ = 12.202, *p* < 0.001), while the incidence of bradycardia requiring intervention was higher in the dexmedetomidine group (*χ*^2^ = 4.345, *p* = 0.034). There was no statistically significant difference in the incidence of other adverse events such as hypotension, nausea, and vomiting between the groups see Table 4.

**Table 4:** Comparison of adverse events between the two groups [*n*(%)].

Adverse event	Propofol group (*n* = 145)	Dexmedetomidine Group (*n* = 150)	*χ*^2^ value	*p*-value
Injection pain	20 (13.79)	4 (2.67)	12.202	<0.001
Hypotension (requiring intervention)	8 (5.52)	6 (4.00)	0.581	0.447
Bradycardia (requiring intervention)	4 (2.76)	13 (8.67)	4.345	0.034
Nausea and vomiting	12 (8.28)	10 (6.67)	0.28	0.597
Dry mouth	5 (3.45)	9 (6.00)	1.053	0.305
New-onset arrhythmia	3 (2.07)	5 (3.33)	0.462	0.497
Hypoxemia (SpO₂ < 90%)	11 (7.59)	7 (4.67)	1.088	0.297
Any adverse event	43 (29.66)	38 (25.33)	0.702	0.402

### 3.6 Multivariable regression analysis

**Figure 3:**
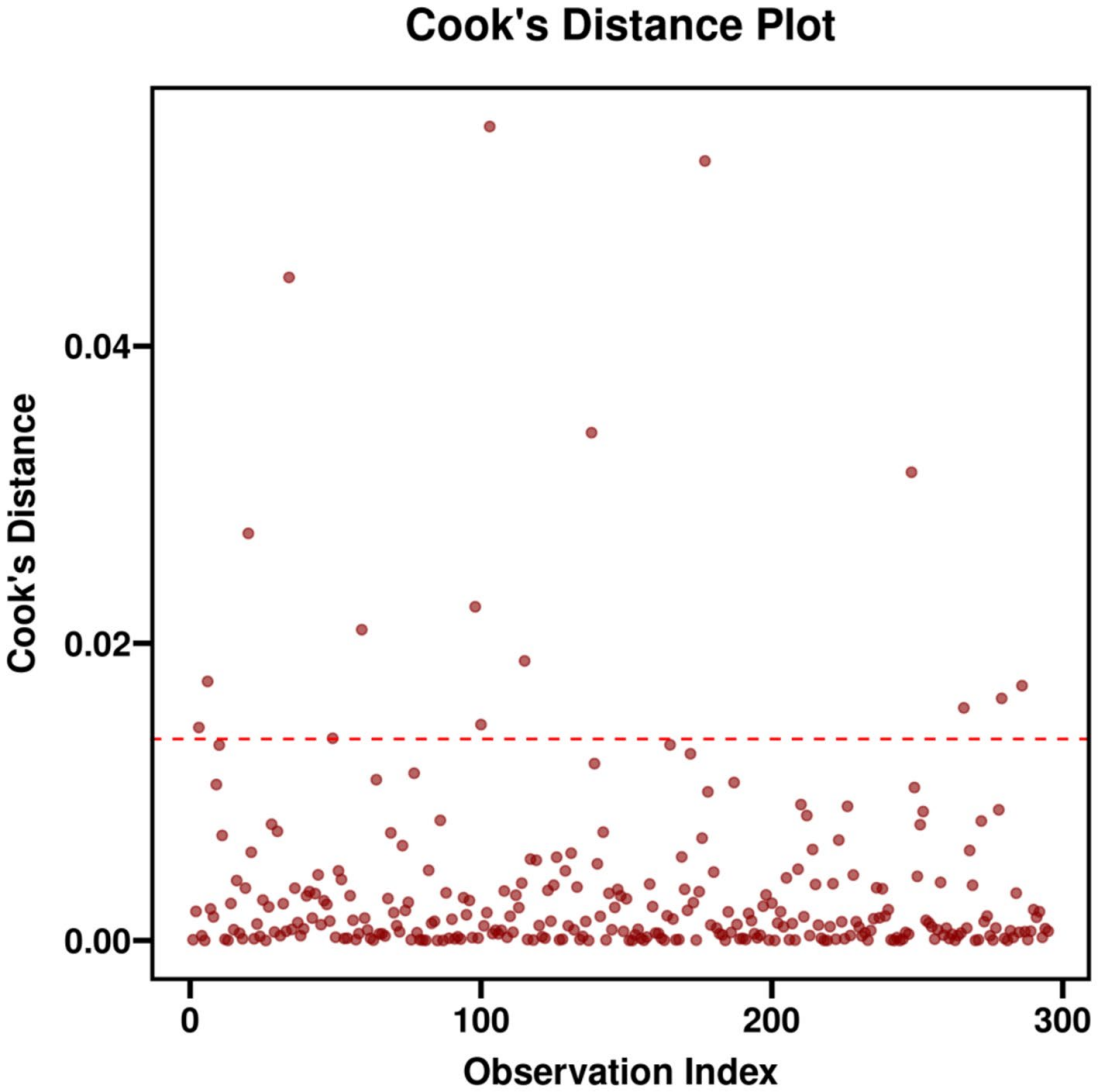
Cook’s distance plot. Cook’s distance plot for the multivariable linear regression model. No influential outliers are present (all values <1).

**Figure 4:**
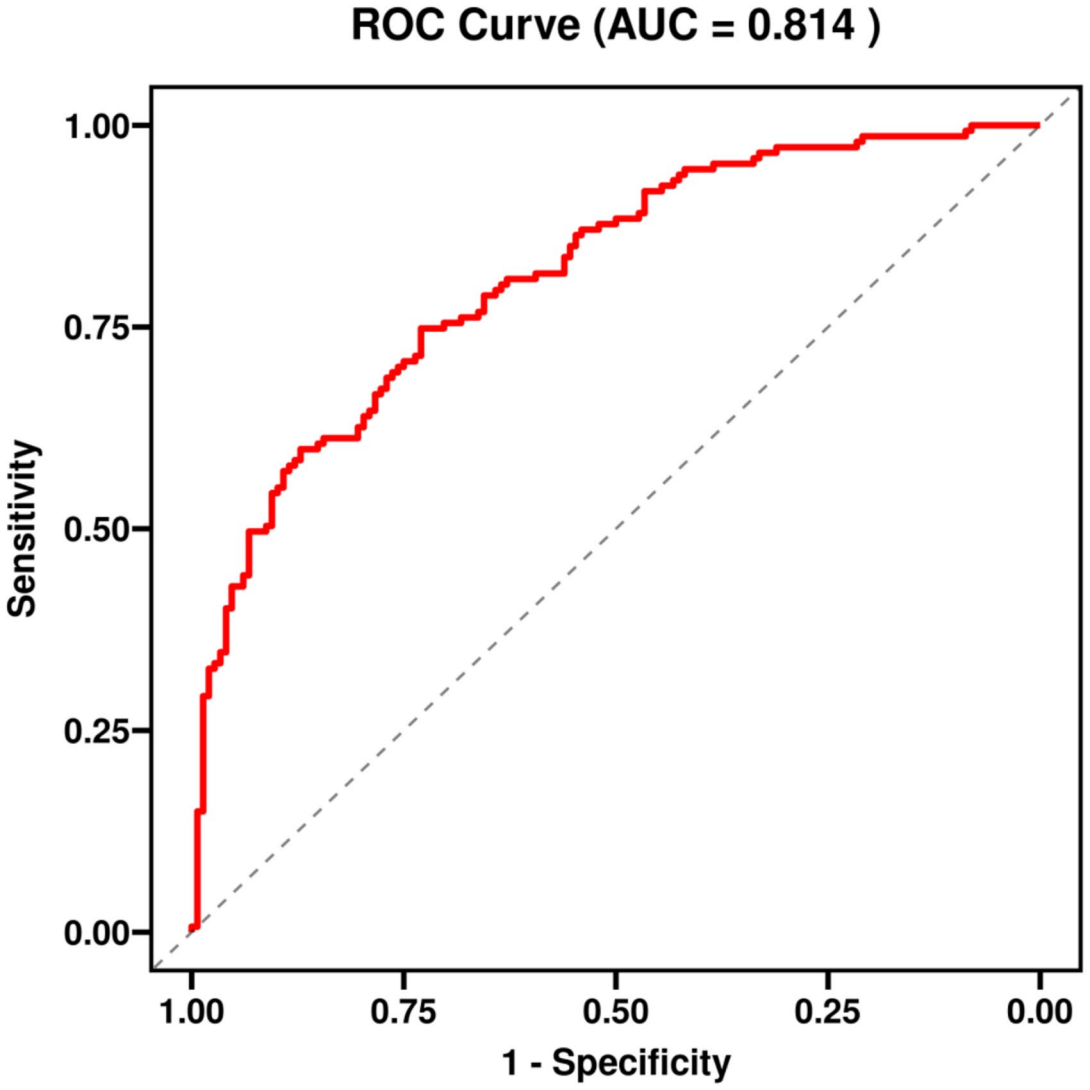
ROC curve.

The multivariate linear regression analysis in Table 5 uses RAVLT delayed recall score as the dependent variable. The results showed that sedative drug selection (dexmedetomidine vs. propofol) was an independent positive predictor (non-standardized *β* = 0.85, *t* = 2.729, *p* = 0.007), preoperative MMSE score was also a positive predictor (non-standardized *β* = 0.42, *t* = 3.125, *p* = 0.002), and age was a negative predictor (non-standardized *β* = −0.05, *t* = −2.372, *p* = 0.018). See Table 5. The multivariable linear regression model demonstrated good overall fit and reliability. Assessment of multicollinearity revealed no concerns, as all variance inflation factors (VIF) were close to 1 (all <1.2). Examination of the Cook’s distance plot (Figure 3) identified no influential outliers, confirming the stability of the regression estimates. Residual diagnostics further supported the adequacy of the model, with no violation of homoscedasticity or normality assumptions. The predictive metric for RAVLT delayed recall was calculated for each patient using the multivariable linear regression equation: Predicted RAVLT delayed recall score = 7.58 + [0.85 × Sedative Agent (Dexmedetomidine = 1, Propofol = 0)] + [−0.05 × Age (years)] + (0.42 × Preoperative MMSE Score) + [−0.01 × Duration of Surgery (min)] + [−0.32 × Sex (Male = 1, Female = 0)]. The area under the ROC curve (AUC) was 0.814 (Figure 4), reflecting excellent discriminative ability and indicating that the model reliably distinguishes between individuals with poor and preserved delayed recall performance.

**Table 5:** Multiple linear regression: predictors of RAVLT delayed recall score.

Independent variable	Unstandardized *β* (95% CI)	Standardized *β*	*t*-value	*p*-value	VIF
Sedative agent (Dexmedetomidine vs. Propofol)	0.85 (0.24, 1.46)	0.143	2.729	0.007	1.026
Age (years)	−0.05 (−0.09, −0.01)	−0.192	−2.372	0.018	1.104
Preoperative MMSE score	0.42 (0.15, 0.69)	0.268	3.125	0.002	1.012
Duration of surgery (min)	−0.01 (−0.03, 0.01)	−0.088	−1.134	0.257	1.045
Sex (Male vs. Female)	−0.32 (−0.98, 0.34)	−0.054	−0.956	0.339	1.038

### 3.7 ANCOVA-adjusted primary outcomes

After adjusting for age, preoperative MMSE score, and duration of surgery, the ANCOVA estimated least-squares mean RAVLT delayed recall score was 8.10 (95% CI 7.58–8.62) in the dexmedetomidine group and 7.22 (95% CI 6.70–7.74) in the propofol group (adjusted mean difference 0.88 points, 95% CI 0.18–1.58, *p* = 0.014). The adjusted picture naming reaction time was 878.3 ms (95% CI 850.2–906.4) in the dexmedetomidine group and 850.6 ms (95% CI 822.6–878.6) in the propofol group (adjusted mean difference 27.7 ms, 95% CI 2.5–52.9, *p* = 0.032). These results are summarized in Table 6.

**Table 6:** ANCOVA-adjusted least-squares means for primary outcomes.

Primary outcome	Group	Adjusted LS mean (95% CI)	Adjusted difference (95% CI)	*p*-value
RAVLT delayed recall score	Dexmedetomidine	8.10 (7.58, 8.62)	0.88 (0.18, 1.58)	0.014
	Propofol	7.22 (6.70, 7.74)		
Picture naming reaction time (ms)	Dexmedetomidine	878.3 (850.2, 906.4)	27.7 (2.5, 52.9)	0.032
	Propofol	850.6 (822.6, 878.6)		

ANCOVA models included age, preoperative MMSE score, and duration of surgery as 0.covariates.

LS mean, least-squares mean; CI, confidence interval.

## 4 Discussion

The impact of intravenous sedatives on postoperative cognitive function is one of the core issues of concern in perioperative neuroscience. Propofol and dexmedetomidine, as the two most commonly used intravenous sedatives in clinical practice, have completely different mechanisms of action (16). Propofol exerts sedative and hypnotic effects by enhancing the inhibitory effect of gamma aminobutyric acid (GABA) receptors, while dexmedetomidine selectively activates central *α* 2-adrenergic receptors, producing a sedative state closer to natural sleep (17). From our data, there is a significant difference in the impact of these two types of drugs on early postoperative neurocognitive function (18). On the first day after surgery, the explicit memory consolidation ability reflected by the Rey Auditory Word Learning Test (RAVLT) delayed recall score in the dexmedetomidine group was significantly better than that in the propofol group (*p* = 0.015), while the implicit memory (image naming reaction time) performance of the two groups showed an opposite trend (19). This result suggests that in clinical practice, the selection of different sedatives may not only focus on hemodynamics or awakening speed, but their differential impact on cognitive function should also not be ignored (20, 21).

The advantage of dexmedetomidine in protecting explicit memory may be related to its specific mechanism of action on the locus coeruleus. Compared to the extensive cortical inhibition caused by propofol, dexmedetomidine can regulate the sleep wake cycle and preserve the integrity of some cognitive circuits by stimulating the locus coeruleus alpha 2 receptor. Previous literature supports that dexmedetomidine can alleviate propofol induced neuronal apoptosis and inflammatory response, providing an explanatory perspective for the observed memory differences (22). A study based on intracranial electroencephalography also found that the EEG activity pattern generated by dexmedetomidine, especially in the delta frequency band and changes in network entropy, is closer to the natural N2 sleep phase compared to propofol (23). This’ more sleep like ‘neural state may be more conducive to memory consolidation (24). However, it is worth noting that although the difference between groups reached statistical significance, the RAVLT score only differed by 0.84 points, and the clinical significance of this magnitude still needs to be explored (25).

In contrast, the dexmedetomidine group showed a significant prolongation in image naming reaction time (*p* = 0.037), suggesting that it may not be as effective as propofol in terms of processing speed and perceptual priming in implicit memory. One possible explanation for this discovery is the sedative effect of residual dexmedetomidine (26). Although we controlled for the time it took for two groups of patients to reach an Aldrete score ≥ 9 after discontinuation, the elimination half-life of dexmedetomidine was longer, and residual pharmacological effects may lead to slower task processing speed (27). A previous study on awake craniotomy also pointed out that in patients receiving compound dexmedetomidine sedation, the reaction time of intraoperative image naming was significantly longer than that of the propofol group alone (28). This suggests that dexmedetomidine may not be the optimal choice in terms of processing speed (29). The evidence in the literature is relatively consistent on this point, indicating that the effect may be more biased towards the inherent pharmacological properties of the drug rather than a simple issue of sedation depth (30).

There are clear differences in the characteristics of awakening quality and hemodynamics between the two groups of drugs. The median time to recovery in the dexmedetomidine group was 18 min, significantly longer than the 14 min in the propofol group (*p* < 0.001), which is similar to previous observational studies. From a safety perspective, the incidence of bradycardia (requiring intervention) in the dexmedetomidine group was significantly higher than that in the propofol group (8.67% vs. 2.76%, *p* = 0.034), while injection pain was more prominent in the propofol group (13.79% vs. 2.67%). The difference in safety profile is fully consistent with their respective pharmacological characteristics: the alpha 2 receptor agonistic effect of dexmedetomidine can cause a decrease in heart rate and cardiac output, while propofol, as an alkylphenol preparation, often causes pain at the injection site. It is worth noting that there was no significant difference in the incidence of hypoxemia or hypotension between the two groups, indicating that the two drugs have roughly equivalent respiratory and circulatory safety—this is consistent with clinical observations in the real world.

Our multivariate regression analysis further confirms that the choice of sedative drugs (dexmedetomidine vs. propofol) is an independent influencing factor for RAVLT delayed recall score (*β* = 0.85, 95% CI 0.24–1.46, *p* = 0.007), while age and preoperative MMSE score are also significant predictive factors. Subgroup analysis did not find any significant covariate modification effects, suggesting that the observed explicit memory differences may have universality across subgroups.

Notably, the dexmedetomidine group showed a lower incidence of early postoperative global cognitive decline as measured by a ≥ 2-point decrease in MMSE score (14.67% vs. 24.14%, *p* = 0.037). This finding suggests that, despite slower processing speed, overall cognitive stability—as captured by a brief screening instrument—may be better preserved with dexmedetomidine. The discrepancy between the MMSE and naming reaction time results likely arises because the MMSE does not assess processing speed, whereas the naming task specifically isolates this domain. These complementary findings reinforce the need for multi-dimensional cognitive evaluation when comparing sedative agents.

The mean difference of 34 ms in picture naming reaction time represents a small effect size (Cohen’s d = 0.28). In clinical practice, this subtle slowing is unlikely to be noticeable or functionally limiting for the majority of patients. Therefore, while this finding suggests a potential pharmacodynamic effect of dexmedetomidine on processing speed, it should not be interpreted as a contraindication for its use; rather, it may inform drug selection in scenarios where utmost cognitive speed is required, such as in patients needing to resume high-demand cognitive tasks immediately after recovery.

## 5 Strengths and limitations

This study has several strengths, including a comprehensive assessment of both explicit and implicit memory, a standardized cognitive evaluation protocol embedded in routine care, and adjustment for key confounders. However, as a retrospective cohort study, the inherent selection bias and residual confounding in this study are difficult to completely eliminate. Although we performed regression correction for multiple known confounding factors, unmeasured factors such as real-time monitoring of specific sedation depth and medication preferences of different anesthesia operators may still affect the outcome. In addition, the sample size was not pre estimated based on the primary outcome, and although post efficacy analysis showed that the reliability of the primary outcome was acceptable, prospective validation with a large sample size is still necessary. Due to the low proportion of missing data (less than 2%) and its random nature, we did not use multiple imputation, which may introduce bias under certain conditions and cannot be ignored. Third, the dosing of sedatives was clinically titrated and covered a wide range, introducing variability that may have influenced the cognitive outcomes. Future studies with more standardized dosing or dose–response analysis are needed. Furthermore, propofol was administered via either target-controlled infusion or manual infusion per clinician preference, which may have contributed to pharmacokinetic variability; however, both modes are standard practice and the effect on our outcomes is likely limited.

In summary, in this retrospective study of short-term intravenous sedation, dexmedetomidine was associated with a marginal advantage in early postoperative explicit memory over propofol, accompanied by a subtle reduction in processing speed. Both effects were small and their clinical relevance remains to be established. The two drugs exhibited distinct safety profiles. Future prospective research is needed to determine whether these pharmacologically plausible differences translate into meaningful long-term functional outcomes and to define patient subgroups that might benefit preferentially from one agent over the other. However, the between-group difference of 0.84 points on the RAVLT delayed recall, although statistically significant, represents a small effect size (Cohen’s d = 0.28) and must be interpreted with caution.

## Data Availability

The original contributions presented in the study are included in the article/supplementary material, further inquiries can be directed to the corresponding author.
